# Supplementary material for: Empirical methods for controlling false positives and estimating confidence in ChIP-Seq peaks
Source: BMC Bioinformatics. 2008 Dec 5;9:523. doi: 10.1186/1471-2105-9-523 (PMC2628906; doi:10.1186/1471-2105-9-523)
Supplement: Additional File 1 — A variety of html documents from the USeq web site detailing the available applications, their best usage, output file type descriptions, command line menus, etc. [file 1471-2105-9-523-S1.zip › USeqUserGuides/JavaDoc.zip!/JavaDoc/util/bio/parsers/Fasta.html]

Fasta


|  |  |  |
| --- | --- | --- |
| **Overview** \| **Package** \| **Class** \| **Use** \| **Tree** \| **Deprecated** \| **Index** \| **Help** | |  |
| **PREV CLASS**   **NEXT CLASS** | **FRAMES**    **NO FRAMES**     **All Classes** |  |
| SUMMARY: NESTED \| FIELD \| CONSTR \| METHOD | DETAIL: FIELD \| CONSTR \| METHOD |  |


---


## util.bio.parsers Class Fasta

```
java.lang.Object
  util.bio.parsers.Fasta
```

**All Implemented Interfaces:**: java.lang.Comparable

---

``` public class Fasta extends java.lang.Object implements java.lang.Comparable ```

---

**Constructor Summary**

`Fasta(java.lang.String name,
java.lang.String seq)`


| **Method Summary** | |
| --- | --- |
| `int` | `compareTo(java.lang.Object obj)` |
| `java.lang.String` | `getName()` |
| `int` | `getNumber()` |
| `java.lang.String` | `getSeq()` |
| `void` | `setName(java.lang.String name)` |
| `void` | `setNumber(int number)` |
| `void` | `setSeq(java.lang.String seq)` |

**Methods inherited from class java.lang.Object**

`equals, getClass, hashCode, notify, notifyAll, toString, wait, wait, wait`

**Constructor Detail**

### Fasta

```
public Fasta(java.lang.String name,
             java.lang.String seq)
```


**Method Detail**

### compareTo

```
public int compareTo(java.lang.Object obj)
```

:   **Specified by:**: `compareTo` in interface `java.lang.Comparable`

---


### getName

```
public java.lang.String getName()
```

---


### setName

```
public void setName(java.lang.String name)
```

---


### getNumber

```
public int getNumber()
```

---


### setNumber

```
public void setNumber(int number)
```

---


### getSeq

```
public java.lang.String getSeq()
```

---


### setSeq

```
public void setSeq(java.lang.String seq)
```


---


|  |  |  |
| --- | --- | --- |
| **Overview** \| **Package** \| **Class** \| **Use** \| **Tree** \| **Deprecated** \| **Index** \| **Help** | |  |
| **PREV CLASS**   **NEXT CLASS** | **FRAMES**    **NO FRAMES**     **All Classes** |  |
| SUMMARY: NESTED \| FIELD \| CONSTR \| METHOD | DETAIL: FIELD \| CONSTR \| METHOD |  |


---
